# Are endocasts reliable proxies for brains? A 3D quantitative comparison of the extant human brain and endocast

**DOI:** 10.1111/joa.13318

**Published:** 2020-09-30

**Authors:** Jean Dumoncel, Gérard Subsol, Stanley Durrleman, Anne Bertrand, Edwin de Jager, Anna C. Oettlé, Zarina Lockhat, Farhana E. Suleman, Amélie Beaudet

**Affiliations:** ^1^ Laboratoire d’Anthropobiologie Moléculaire et Imagerie de Synthèse UMR 5288 CNRS Université Toulouse 3 Paul Sabatier Toulouse France; ^2^ Research‐Team ICAR Laboratoire d’Informatique de Robotique et de Microélectronique de Montpellier CNRS Université de Montpellier Montpellier France; ^3^ Aramis team INRIA Paris Sorbonne Universités UPMC Université Paris 06 UMR S 1127 Inserm U 1127 CNRS UMR 7225 Institut du Cerveau et de la Moelle épinière Paris France; ^4^ Department of Neuroradiology Hôpital Pitié‐Salpêtrière Assistance Publique–Hôpitaux de Paris Paris France; ^5^ Department of Anatomy Faculty of Health Sciences University of Pretoria Pretoria South Africa; ^6^ Department of Anatomy and Histology School of Medicine Sefako Makgatho Health Sciences University Ga‐Rankuwa South Africa; ^7^ Department of Radiology Faculty of Health Sciences University of Pretoria Pretoria South Africa; ^8^ Department of Archaeology University of Cambridge Cambridge United Kingdom; ^9^ School of Geography, Archaeology and Environmental Studies University of the Witwatersrand Johannesburg South Africa

**Keywords:** automatic segmentation, brain shape, paleoneurology, sulci, surface‐based comparison

## Abstract

Endocasts (i.e., replicas of the inner surface of the bony braincase) constitute a critical proxy for qualifying and quantifying variations in brain shape and organization in extinct taxa. In the absence of brain tissues preserved in the fossil record, endocasts provide the only direct evidence of brain evolution. However, debates on whether or not information inferred from the study of endocasts reflects brain shape and organization have polarized discussions in paleoneurology since the earliest descriptions of cerebral imprints in fossil hominin crania. By means of imaging techniques (i.e., MRIs and CT scans) and 3D modelling methods (i.e., surface‐based comparisons), we collected consistent morphological (i.e., shape) and structural (i.e., sulci) information on the variation patterns between the brain and the endocast based on a sample of extant human individuals (N = 5) from the 3D clinical image database of the Steve Biko Academic Hospital in Pretoria (South Africa) and the Hôpitaux Universitaires Pitié Salpêtrière in Paris (France). Surfaces of the brain and endocast of the same individual were segmented from the 3D MRIs and CT images, respectively. Sulcal imprints were automatically detected. We performed a deformation‐based shape analysis to compare both the shape and the sulcal pattern of the brain and the endocast. We demonstrated that there is close correspondence in terms of morphology and organization between the brain and the corresponding endocast with the exception of the superior region. By comparatively quantifying the shape and organization of the brain and endocast, this work represents an important reference for paleoneurological studies.

## Graphical abstract

Is the endocast a reliable proxy for reconstructing the brain of fossil specimens? By quantitatively comparing extant human brains and endocasts using MRIs and CT‐scans, here we address this fundamental question and support the significance of fossil endocasts in paleoneurological studies.

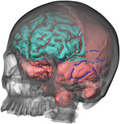

## 1 INTRODUCTION

In the absence of fossilized brains, reconstructing human brain evolutionary history is particularly challenging. Paleoneurological evidence primarily relies on the interpretation of fossil endocasts, which represent replicas of the inner table of the bony braincase. Endocasts provide the only direct evidence of brain evolution in extinct taxa and constitute a critical proxy for qualifying and quantifying variations in brain size, shape and organization throughout human evolution (Bruner, 2015; Bruner et al., 2018; Falk, 2014; Holloway, 1978; Holloway et al., 2004; Neubauer, 2014; Zollikofer and Ponce de León, 2001, 2013). However, the correspondence of the shape of the brain to the shape of the endocast, as well as the correlation of the gyral and sulcal pattern in the brain external surface with the bulges and furrows imprinted on the inner surface of the braincase, have been the focus of major historical debates.

Following the pioneer descriptions of the fossil endocasts of *Pithecanthropus erectus* from Java (Dubois, 1898) and of the Neanderthal specimen from La Chapelle‐aux‐Saints (Boule and Anthony, 1911), Symington (1916) severely criticized the identification of brain imprints in the endocast, stating “That the simplicity or complexity of the cerebral fissures and convolutions cannot be determined with any degree of accuracy from endocranial casts, even on complete skulls, much less on reconstructions from imperfect skulls.” (p. 130). In their reply, Boule and Anthony (1917, p. 96) expressed that “It would be regrettable in every respect if we should refuse to avail ourselves in paleontology of the endocranial casts”. Later on, following the discovery of the Taung child (Dart, 1925), Le Gros Clark et al. (1936) compared six chimpanzee crania and their corresponding brains based on post‐mortem observations. Despite their conclusion that “very little information can be extracted in regard to sulcal pattern from the majority of our endocranial casts of the chimpanzee” (p. 267), they reported the identification of crucial sulci, such as the fronto‐orbital sulcus delimitating the orbital cap. More recently, similar investigations performed on a sample of macaques revealed that the locations of most of the cerebral sulci could be inferred from the inner surface of the cranium (Kobayashi et al., 2014).

In this context, quantifying the degree of reliability of the endocast in order to enable further credible discussion of brain evolutionary changes in the fossil record is of prime interest. The development of advanced imaging techniques (e.g., magnetic resonance imaging abbreviated as MRI, computed‐tomography abbreviated as CT) and analytical methods (e.g., geometric morphometrics) in neurosciences and paleoneurology offers a unique opportunity to address this long‐standing question by applying innovative comparative quantitative methods. More specifically, in the last decades, software that are now widely used in neurosciences have been developed for virtually manipulating, automatically segmenting (e.g., regional segmentation of the brain), identifying and analysing neuroanatomical features in brains from volumetric image data (e.g., Borne et al., 2020; Reuter et al., 2012; Rivière et al., 2009). Similarly, automatic segmentation methods for generating virtual endocasts are now available in paleosciences (e.g., Endex, Endomarker, Michikawa et al., 2017; Profico et al., 2020; Subsol et al., 2010). However, analytical tools for the automatic recognition and identification of cerebral imprints in endocasts are still scarce (e.g., automatic detection of sulcal imprints, Beaudet et al., 2016, 2019a; de Jager et al., 2019).

Directly comparing the brain and the endocast is technically challenging. First, such studies require a posteriori MRIs and CT scans of the same non‐pathological living individual. Even if some studies demonstrated that bone tissues could be visualized using MRIs, images collected using this modality are not suitable for accurately reconstructing the fine structural aspects of the braincase such as brain imprints (Dogdas et al., 2005). Second, the level of details in 3D reconstructions of brain tissues using CT scans is not sufficient for characterizing brain circumvolutions (e.g., Figure 2 in Irimia et al., 2019). Moreover, since some cortical structures may not be systematically found in the endocast (see de Jager et al., 2019), the methodology developed for comparing the brain and the corresponding endocast should be applicable to partial data (i.e., incomplete sulcal patterns).

To the best of our knowledge, the studies of Zollikofer and Ponce de León (2001) represents the first attempt for mapping potential shape differences/similarities between the brain and the endocast. Although they suggested “marked deviations” between the shape of the brain and the endocast, they were not able to compare major sulcal imprints (i.e., underrepresentation of the prominent cortical structures), which might be explained by the relatively high values of the resolutions of the MRIs and CT scans used (i.e., up to 4 mm), and the inability of their technical approach to detect these features (i.e., brain‐to‐endocast distances) and their limited sample (i.e., two individuals). More recently, Fournier et al. (2011) further investigated brain to endocast distances in 37 individuals and demonstrated that the endocast shows the same asymmetry pattern as the brain, thus proving the relevance of the endocast for tracking changes in the asymmetry of the brain. Finally, the recent study of Alatorre Warren et al. (2019) addressed the issue of covariation between brain and neurocranial features in extant humans and chimpanzees but did not provide a direct comparison of the brain and the endocast (see also Albessard, 2018). Accordingly, to date, the degree of reliability of the endocast for identifying key cerebral aspects remains largely unknown.

Here, we provide a combined analysis of the brain and the corresponding endocast of the same extant human individuals (N = 5) by using multimodality imaging techniques (i.e., MRIs and CT scans) for quantitatively assessing the degree of reliability of the endocast in paleoneurological studies. More specifically, our study focuses on the comparative study of the morphology, which means the global (i.e., entire volume) and regional (i.e., lobes) shape, and of the structure, which means the position and spatial relationships of the sulci, of the brain and the endocast.

## 2 MATERIALS AND METHODS

### 2.1 Materials

We collected MRIs and CT scans of a total of five extant human individuals from the clinical record of the Steve Biko Academic Hospital in Pretoria (South Africa) (N = 4) and the *Hôpitaux Universitaires Pitié Salpêtrière* in Paris (France) (N = 1) of known age ranging from 30 to 69 years old (Table 1). Data were collected a posteriori between 2016 and 2019. We systematically excluded any individuals with pathologies affecting the brain and/or the braincase. Spatial resolution of MRIs and CT scans varies from 0.375 to 2 mm (Table 1). Concerning the MRIs, multiple sequences were used, including T1‐weighted (produced by using short time to echo and short repetition time) and T2‐weighted (produced by using long time to echo and long repetition time). Additionally, infusion of gadolinium and flair (i.e., fluid attenuated inversion recovery with very long time to echo and very long repetition time) were used. All of the patients included in our study lay supine during the acquisition process.

**Table 1:** List of individuals included in the study. F: female; HUPS: *Hôpitaux Universitaires Pitié Salpêtrière*; M: male; SBAH: Steve Biko Academic Hospital

Specimens	Sex	MRI resolution (mm)	CT scan resolution (mm)	MRI sequences	Source
I1	M	0.833 × 0.833 × 1.000	0.488 × 0.488 × 0.500	T1‐weighted	SBAH
I2	M	0.744 × 0.744 × 2.000	0.488 × 0.488 × 0.500	T1‐weighted GAD	SBAH
I3	F	0.756 × 0.756 × 1.000	0.457 × 0.457 × 1.000	T1‐weighted GAD	SBAH
I4	F	0.700 × 0.547 × 0.547	0.375 × 0.375 × 0.625	T2‐weighted Flair	HUPS
I5	F	0.744 × 0.744 × 2.000	0.449 × 0.449 × 0.500	T1‐weighted GAD	SBAH

### 2.2 Methods

**Figure 1:**
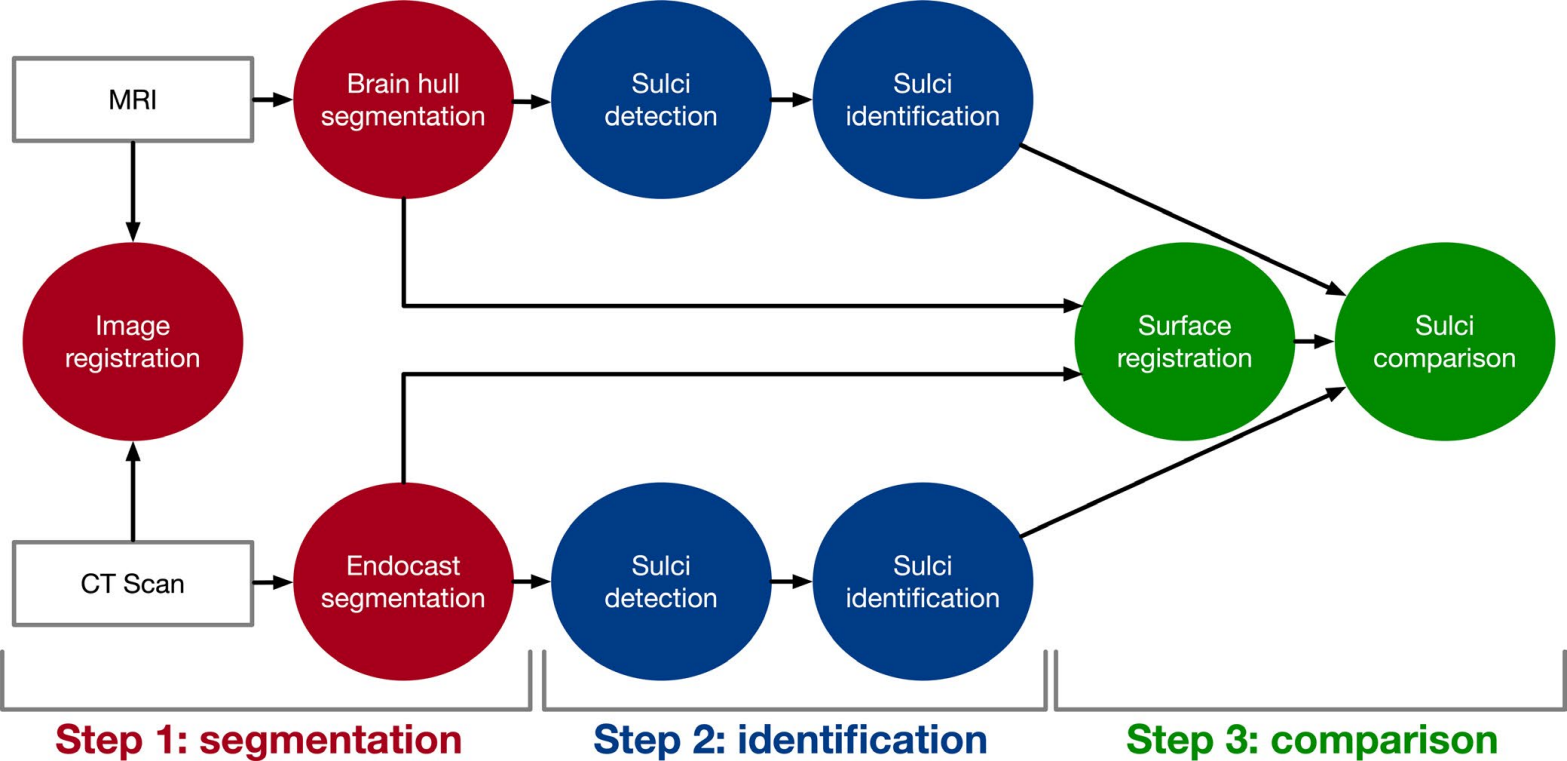
Workflow of the comparative analysis of the brain and the endocast

We defined a workflow to virtually generate, identify and analyse the shape and organization of the brain and endocast from the MRIs and CT scans (Figure 1). Our workflow could be summarized as follows: first, the images from the MRI and CT scan acquisitions were registered for a preliminary observation of the correspondence between the brain and the cranium and the brain hull and the endocast were segmented from the MRIs and CT scans, respectively (step 1); second, sulci were detected and identified on the brain hull and endocast (step 2); finally, the shape of the brain hull and of the endocast, as well as the sulcal patterns of the brain hull and endocasts, were directly compared using deformation (step 3).

#### 2.2.1 Step 1: Image registration and segmentation of the brain hull and the endocast

**Figure 2:**
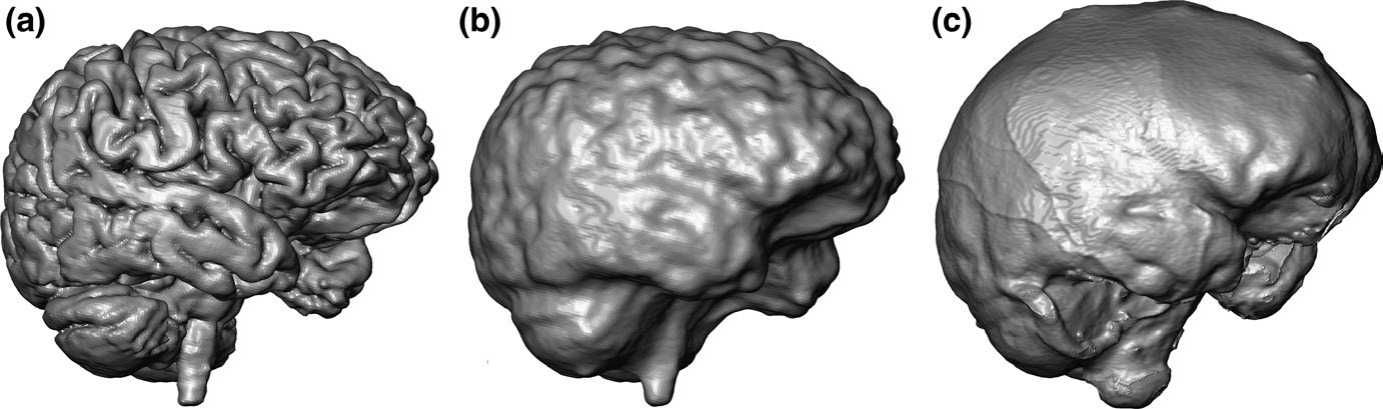
Virtual rendering of the external surfaces of the brain (a), the brain hull (b) and the endocast (c) of the same individual (I1)

**Figure 3:**
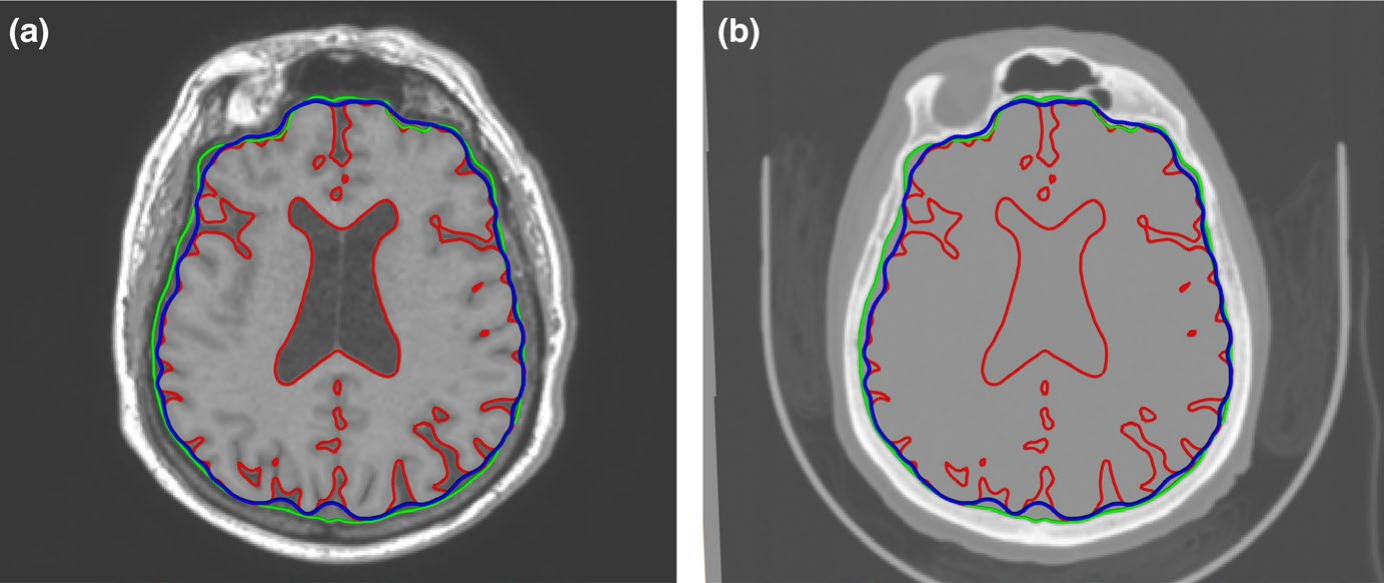
Registration of the images from the MRIs (a) and CT scans (b) with the segmentation of the brain (red, generated from the MRIs and transferred to the CT scans), brain hull (blue, generated from the MRIs and transferred to the CT scans) and endocast (green, generated from the CT scans and transferred to the MRIs) being superimposed. The patient lays supine

A similarity measure using a normalized mutual information metric on Avizo v8.0 (Visualization Sciences Group Inc.) was used for the initial registration of MRIs and CT scans so that they could be visually compared. The brain was automatically segmented from MRIs using the software BrainVISA and Morphologist (Rivière, 2009) (Figure 2). Since the topography of the external cortical surface is more complex than the external surface of the endocast, we used the brain hull that is a simplified yet accurate representation of the brain surface. However, the simplification operation was visually and manually checked to ensure that enough details (i.e., sulci) are preserved. We thus generated a brain hull by using a sphere that encompassed the brain and that was deformed following an iterative process using Endex software (Figure 2; Subsol, 2010). The endocast was similarly segmented from the CT scans using a sphere placed inside the braincase and deformed following an iterative process. Further virtual cleaning was performed using Avizo v8.0. Thereafter the brain hull and the endocast were represented as 3D meshes, which were resampled to 100,000 triangular faces. Results of the alignment and segmentation processes are shown in Figure 3.

#### 2.2.2 Step 2: Detection and identification of the brain and endocast sulci

Sulci from the brain hull and the endocast were detected using an automatic method that is based on the algorithm introduced by Yoshizawa et al. (2008) for the detection of topographical variations (i.e., ridge and ravine lines) in 3D meshes (Beaudet et al., 2016, 2019a; Beaudet and Gilissen, 2018; de Jager et al., 2019). Since sulci could be considered to be the salient parts of the brain hull surface, these structures could be detected via a differential geometry‐based approach. Accordingly, at each point of the 3D mesh, the principal curvatures can be computed and the sulci would then correspond to some of their extrema (Subsol, 1999).

Sulci from the brain hull and the endocast were manually identified using a MATLAB R2013a v8.1 (Mathworks) program (https://gitlab.com/jeandumoncel/curve‐editor; Beaudet et al., 2016, 2019a; de Jager et al., 2019) and endocast atlases from previous publications (Connolly, 1950; de Jager et al., 2019). A label represented by a colour was attributed to each category of sulci. As observed in brain and endocast atlases, sulci might be incomplete or split into several fragments. Identified sulci could thus be represented by a curve or a group of curves.

#### 2.2.3 Step 3: Surface and sulci comparisons

Shape analysis of the brain hull and endocast was performed using the software Deformetrica v4 written in Python for the statistical analysis of 3D shape data (Durrleman et al., 2014). After the first superimposition of the images (step 1 in section 2.2.1) that aims at visually comparing the CT and MRI data is performed (section 2.2.1), a second superimposition process is computed to compare the shape of the endocast and the brain hull using a rigid alignment and uniform scaling via the “Align surface” tool in Avizo. The surface of the brain hull was then deformed to the surface of the endocast using a process known as registration via Deformetrica v4 (Durrleman et al., 2014). The registration parameters were set to include a large number of control points (about 100,000 points) in order to be able to define a very complex deformation so that the brain hull could closely match the endocast.

**Figure 4:**
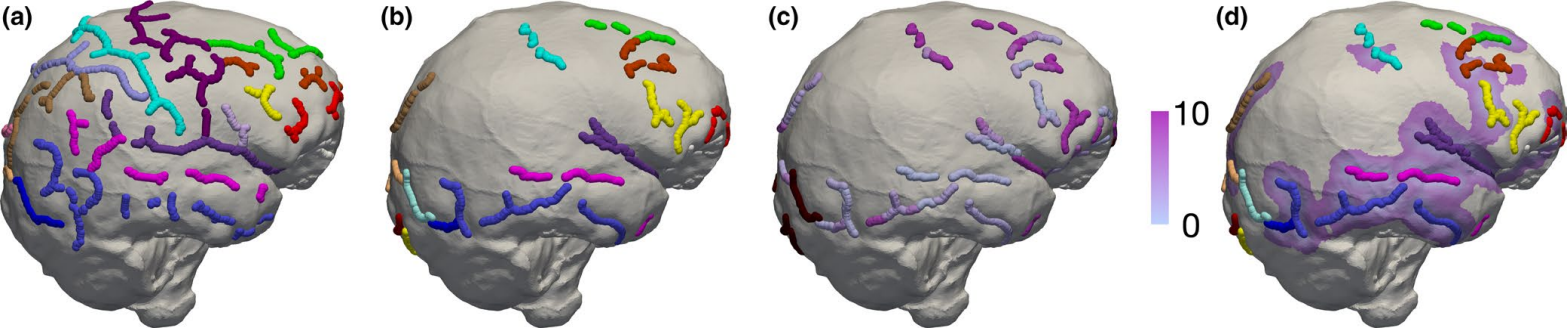
Comparison of the location of the sulci in the brain hull (a) and endocast (b) once the deformation of the brain hull to the endocast is performed and applied to the sulci. Sulci are represented in different colours depending on their label (see Figure 6). Distances of the sulci from the endocast to the brain hull are represented by a colour scale in which light purple corresponds to a close distance and dark purple corresponds to the maximum distance of 10 mm (c). Sulci on the endocast that have not been found on the brain are rendered in dark red in (c). The colour code is extrapolated in (d)

**Figure 5:**
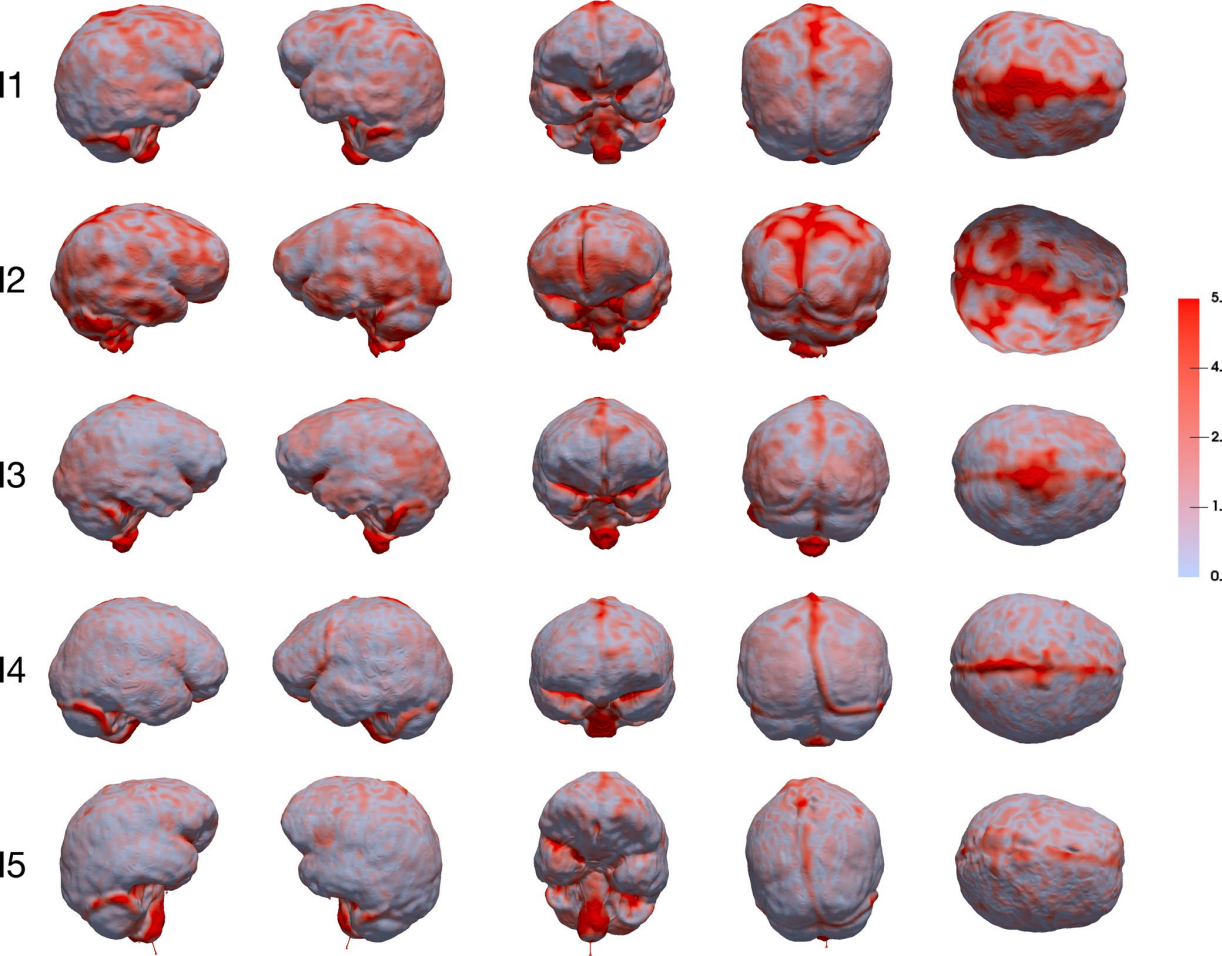
Surface‐based comparison of the brain hull and the endocast. Displacements are rendered by a colour scale ranging from light blue (0 mm) to dark red (5 mm). Endocasts are shown (from left to right) in lateral right, lateral left, anterior, posterior and superior view

Deformation from the brain hull to the endocast was applied to the sulci detected and identified in the brain. Through this process, the sulci detected and identified in the endocast could be directly compared to those from the brain hull. We computed the distance between the curves of the endocast and the corresponding curves on the deformed brain hull. We defined the distance between a curve on the endocast and a curve on the deformed brain hull as follows: for each curve on the endocast, we computed all the distances between each point of this curve and the closest point of the corresponding curves on the deformed brain hull with the same label, and the distance corresponds to the mean of these distances. We consider that the corresponding sulcus in the brain is found when this mean distance is less than 10 mm away from the sulcus detected and identified in the endocast (Figure 4), since this threshold roughly corresponds to the maximum distance between two neighbouring sulci. Thus, sulci that are not associated to any colour maps (for example the retro‐calcarine sulcus in I1, Figure 5) are only represented in endocasts and could not be found on brain hulls or are too far away from each other (i.e., >10 mm). The mean distance of the curves from the endocast to the brain hull was extrapolated so that the differences between the brain hull and endocast sulci could be mapped onto the endocast. We assessed the following: (1) the total number of curves identified in the brain hull (TC‐B) and in the endocast (TC‐E), (2) the number of curves identified in both the endocast and the brain hull at a distance of less than 10 mm from each other (NC‐EB), (3) the number of curves identified in the endocast that has no corresponding identified curves in the brain hull (NS‐EC), and (4) the total number of sulci identified in the brain hull (NS‐B) and in the endocast (NS‐E).

## 3 RESULTS

### 3.1 Comparison of the shape of the brain hull and of the endocast

Figure 5 indicates the displacements, rendered by a pseudo‐colour scale, from the brain hull to the endocast. The maximum value of the colour bar (5 mm) is considered to be the most appropriate compromise representation of both global and local deformations. Even if a certain degree of inter‐individual variation is noticeable (especially between I1, I2 and the rest of the sample), in most of the individuals the temporal and cerebellar lobes as well as the inferior portion of the parietal and frontal lobes of the brain hull are relatively close to the endocast with less than 2 mm differences. Interestingly, the frontal inferior lobule (where the Broca's cap is located) is similar in terms of shape between the brain hull and the endocast. On the contrary, the shape of the superior part of the brain hull differs from the shape of the superior part of the endocast, particularly along the sagittal sinus. The occipital lobes of the brain hull are relatively similar to the corresponding regions in the endocast in I1, I4 and I5, while in I2 and I3 the colour map shows differences.

### 3.2 Comparison of the sulci in the brain hull and the endocast

Table 2 presents the number of curves and sulci identified in the brain hull and in the endocast. In general, the number of curves (TC‐B) and sulci (NS‐B) identified in the brain hull is slightly higher than the number of curves (TC‐E) and sulci (NS‐E) detected in the endocast. Over 70% (with a mean of 80%) of the curves detected in the endocast are found in the brain hull (at a distance of less than 10 mm from the corresponding curves in the brain hull) while less than 6% (with a mean of 4%) of the curves are found only in the endocast.

**Table 2:** Comparison of the curves and sulci detected and identified in the brain hull and the endocast of the five individuals

Specimens	TC‐E	NS‐E	TC‐B	NS‐B	NC‐EB	NS‐EB
I1	139	15	149	17	110	10
I2	169	18	153	17	118	23
I3	178	16	193	19	165	5
I4	152	18	165	17	118	16
I5	249	17	174	18	204	15
Mean	178	17	167	18	143	14

NC‐EB: number of curves identified in both the endocast and the brain hull at a distance of less than 10 mm; NS‐EB: number of curves identified in the endocast that has no corresponding identified curves in the brain hull; TC‐B: total number of curves identified in the brain hull; TC‐E: total number of curves identified in the endocast; NS‐B: total number of sulci identified on the brain hull; NS‐E: total number of sulci identified on the endocast.

**Figure 6:**
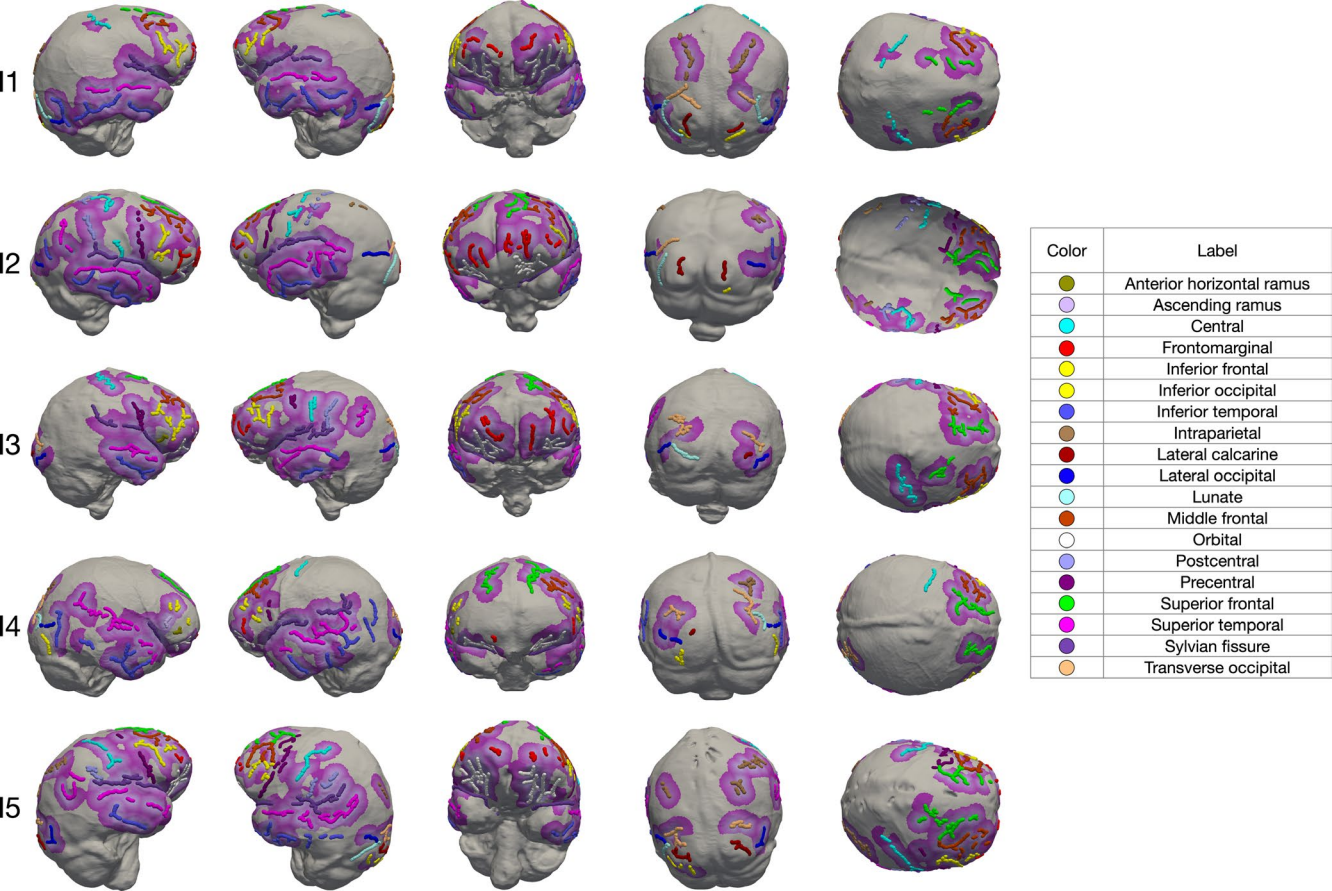
Comparison of the location of the sulci in the brain hull and endocast once the deformation of the brain hull to the endocast is performed and applied to the sulci. Distances between the sulci from the hull transported to the endocast and the corresponding sulci in the endocast are represented by a colour scale in which light purple corresponds to a close distance and dark purple corresponds to the maximum distance of 10 mm. Beyond 10 mm, we consider that the corresponding sulcus on the brain has not been found. Endocasts are shown (from left to right) in lateral right, lateral left, anterior, posterior and superior views

Figure 6 shows the location of the sulci in the brain hull and endocast once the deformation of the brain hull to the endocast is performed and applied to the sulci. Sulci identified in both the endocast and the brain hull are mainly located in the frontal and temporal lobes and, in a minor extent, in the parietal occipital lobes. More specifically, the orbital sulcus, the superior, middle and inferior frontal sulci, the superior, middle and inferior temporal sulci, the Sylvian fissure, the fronto‐marginal sulcus, the precentral, central, and postcentral sulci, the lateral calcarine sulcus, the lateral and transverse occipital sulci and the lunate sulcus are systematically found in the endocast and the brain hull of the five individuals. The ascending and anterior horizontal rami of the Sylvian fissure are found in some individuals (i.e., I2, I3 and I4). Interestingly, the position of the sulci from the endocast is relatively close to the original position in the brain hull except for the occipital lobes and the lunate sulcus, the transverse and inferior occipital sulci and lateral calcarine sulcus are either found far from the original location in the brain (i.e., greater than 10 mm) or were not identified in the brain hull.

## 4 DISCUSSION

In this study, we provide the first direct quantitative comparison of the brain and the endocast of the same extant human individuals that considers both the morphology (i.e., shape) and the structure (i.e., sulcal pattern). While our results suggest a close relationship between the shape and the sulcal pattern of the frontal, temporal and occipital lobes as well as in the inferior portion of the parietal lobes, the correspondence in terms of morphology and organization between the superior part of the brain and of the endocast is more questionable. Nonetheless, our results demonstrate that the morphoarchitecture of critical areas located in the frontal, temporo‐parietal and occipital regions, such as the Broca's cap (located in the inferior region of the frontal lobes), the Wernicke's area (located at the posterior end of the temporal lobes) or the visual cortex (as defined by the lunate sulcus in the occipital lobes), could be inferred from the study of the endocast and contribute to the reconstruction of the chronology and evolutionary process of the hominin brain reorganization (rev. in Beaudet et al., 2019b).

Methodological limitations should be considered as potential factors explaining observable discrepancies between the brain and the endocast. Specifically, the lack of correspondence between the superior regions in the brain hull and in the endocast might be due to potential geometrical distortions in MRIs (Seibert et al., 2016). Moreover, the absence in the brain hull of some of the sulci identified in the endocast could be related to variable spatial resolutions between the MRIs and CT scans, which means that fine features might be detected in the CT scans with a better spatial resolution. Furthermore, when the sulcus is fragmented or incomplete, different fragments of the same sulcus may be identified in the brain hull and in the endocast, thus creating a potential limit in the comparison. Our limited sample, that is explained by the difficulty of collecting MRIs and CT scans of the same non‐pathological individuals, might represent an additional limitation, since that potential sex‐ and age‐related variation cannot be appropriately explored nor assessed. Finally, the presence of the cerebrospinal fluid, of structures related to the brain vascular system and the effect of muscles attached to the cranial vault represent additional factors that may explain the lack of correspondence in some regions of the endocast (Zollikofer and Ponce de León, 2001).

As compared to the landmark study of Zollikofer and Ponce de León (2001), our analysis of the brain and corresponding endocast could identify and compare major sulcal imprints in the brain and the corresponding endocast. Moreover, we did not find any correlations between the topographical distribution of the disparities between the brain and the endocast and the location of large sulci, which may be interpreted in the aforementioned study as an artefact related to the low quality of the images used. Even if their analysis primarily focused on the asymmetries, Fournier et al. (2011) noticed that the brain to endocast distance was greater on the top of the brain/endocast as compared to the bottom and on the front as compared to the back. While our study supports the looser correspondence of the superior region of the endocast relative to the brain, here we did not observe substantial differences between the frontal and occipital lobes, which might be explained by our deformation‐based approach as opposed to their distance‐based approach. Finally, our results temper the conclusion by Alatorre Warren et al. (2019) that “inferences about brain structure cannot and should not be carried out from endocranial shape unless they are accompanied by clear sulcal imprints” since we have demonstrated that certain brain morphologies can indeed be extrapolated from the endocast morphology.

By quantifying and mapping the degree of reliability of endocranial regions, our study provides critical evidence supporting the invaluable contribution of the brain imprints left on the fossil endocranial surfaces to our understanding of the human brain evolutionary history and for discussing key cerebral aspects in the fossil record. Future analyses will be needed to determine if these conclusions also apply to other living or fossil taxa (e.g., Jirak and Janacek, 2017; Watanabe et al., 2018).

## CONFLICT OF INTEREST

The authors declare no conflict of interest.

## AUTHORS’ CONTRIBUTION

Designed/performed research: J.D., A.Bea., E.J., F.S., Z.L., A.O.; contributed new reagents/analytical tools: J.D., G.S., S.D.; collected samples: F.S., Z.L., A.Ber.; analysed/interpreted data: J.D., A.Bea., E.J., G.S., S.D.; wrote/revised the paper: J.D., A.Bea., G.S., S.D., E.J., A.O., F.S., Z.L.

## Data Availability

Deformetrica (https://www.deformetrica.org/) and Endex (https://perso.liris.cnrs.fr/gilles.gesquiere/wiki/doku.php?id=endex) are freely accessible online. The tool we developed for the automatic detection of the sulcal imprints is freely available at: https://gitlab.com/jeandumoncel/curve‐editor.
